# The circadian regulator BMAL1 programmes responses to parasitic worm infection via a dendritic cell clock

**DOI:** 10.1038/s41598-018-22021-5

**Published:** 2018-02-28

**Authors:** Thomas W. Hopwood, Sarah Hall, Nicola Begley, Ruth Forman, Sheila Brown, Ryan Vonslow, Ben Saer, Matthew C. Little, Emma A. Murphy, Rebecca J. Hurst, David W. Ray, Andrew S. MacDonald, Andy Brass, David A. Bechtold, Julie E. Gibbs, Andrew S. Loudon, Kathryn J. Else

**Affiliations:** 1 Faculty of Biology, Medicine and Health, University of Manchester, Oxford Road, Manchester, M13 9PT UK; 2 Manchester Academic Health Sciences Centre, Manchester, United Kingdom; 3 Manchester Collaborative Centre for Inflammation Research, University of Manchester, Manchester, M13 9NT UK

## Abstract

Resistance to the intestinal parasitic helminth *Trichuris muris* requires T-helper 2 (T_H_2) cellular and associated IgG1 responses, with expulsion typically taking up to 4 weeks in mice. Here, we show that the time-of-day of the initial infection affects efficiency of worm expulsion, with strong T_H_2 bias and early expulsion in morning-infected mice. Conversely, mice infected at the start of the night show delayed resistance to infection, and this is associated with feeding-driven metabolic cues, such that feeding restriction to the day-time in normally nocturnal-feeding mice disrupts parasitic expulsion kinetics. We deleted the circadian regulator BMAL1 in antigen-presenting dendritic cells (DCs) *in vivo* and found a loss of time-of-day dependency of helminth expulsion. RNAseq analyses revealed that IL-12 responses to worm antigen by circadian-synchronised DCs were dependent on BMAL1. Therefore, we find that circadian machinery in DCs contributes to the T_H_1/T_H_2 balance, and that environmental, or genetic perturbation of the DC clock results in altered parasite expulsion kinetics.

## Introduction

Parasitic helminthic infection afflicts approximately one third of the world’s population, with the bulk of infection occurring within the gastrointestinal tract^1^. Parasite recognition is mediated by pathogen recognition receptors (PRRs), the triggering of which results in activation of antigen-presenting dendritic cells (DCs), which stimulate and polarise antigen-specific T-cells. The PRRs important in the recognition of *Trichuris* antigens remain undefined. Typically, effective responses to gastrointestinal helminth parasites are mediated by T_H_2-polarised immune responses, which confer protection, but also promote pathologic responses associated with allergic inflammation. Multiple factors are capable of promoting polarised T_H_ cell responses *in vivo*, including cytokine signals, antigen dose, antigen affinity and epigenetic changes^2^, and the nature and relative importance of each may differ according to the infectious agent^3,4^. *Trichuris muris* (*T*. *muris*) is a caecal dwelling parasitic helminth of mice. Expulsion of this parasite has been rigorously characterised and critically requires a dominant T_H_2 immune response. Thus, murine *T*. *muris* infection provides a useful model system for identifying the drivers of T_H_2 responses^5^, with changes in the T_H_1/T_H_2 balance easily monitored via changes in the kinetics of worm expulsion and levels of T_H_2-controlled parasite-specific IgG1 versus T_H_1-controlled IgG2c levels. DC-derived IL-12 is critical for promotion of T_H_1 polarization^6^. However, although IL-4 is a master cytokine in driving T_H_2 immunity, the early cellular sources of IL-4 in helminth infection are less well defined and may involve accessory cells^7–11^. Thus, despite the unequivocal importance of dominant T_H_2 immune responses in immunity to *T*. *muris* infection, the underlying interplay between the DC and T cell remains incomplete.

The circadian clock plays a central role in the regulation of physiological pathways^12^. Cellular oscillations are driven by a complex interplay of cell-intrinsic clock genes, which are linked, to output pathways in a tissue-specific manner^13^. A significant proportion of the expressed transcriptome within tissues is circadian-regulated with reports of up to 43% of protein-coding gene transcripts oscillating in at least one organ^14^. Circadian rhythms play an important role in the regulation of immune function^15^. Both monocytes of the innate immune system and cells of the adaptive immune system such as T and B cells possess components of the circadian clock machinery, which generate autonomous oscillations *in vivo*^16–24^. Further, trafficking and migration of immune cells is also circadian regulated, with daily oscillations in lymphocytes in blood^19,25–27^, T-helper-17 (T_H_-17) cell differentiation^28^, and lymphocyte trafficking through the lymph node^29,30^. Further, in the context of intracellular protozoan parasite infection, circadian regulation of the magnitude of Leishmania infection has recently been described with an underpinning mechanism, thought to involve circadian control of chemokine expression by macrophages and thus rhythmic infiltration of neutrophils and macrophages to the site of infection^31^.

It is not known whether the circadian clock plays a role in immunity to large, extracellular, multicellular parasites such as gastrointestinal helminths. Here, we show that mice infected with *T*. *muris* demonstrate different T_H_ cell responses and consequently different levels of effective parasite expulsion several weeks later, dependent of time-of-day of infection. We show that antigen-presenting DCs are key components; with disruption of time-of-day responses observed in mice with a DC-conditional knock-out of the circadian regulating *Bmal1* gene. Circadian-synchronised DCs *in vitro* showed strongly polarised responses at peak and trough of the circadian cycle, and these were disrupted in *Bmal1*-deficient cells. Collectively our data reveal that a DC circadian clock regulates efficient expulsion of the parasitic nematode *T*. *muris*.

## Results

### *T*. *muris* expulsion kinetics depends on time-of-day of infection

**Figure 1:**
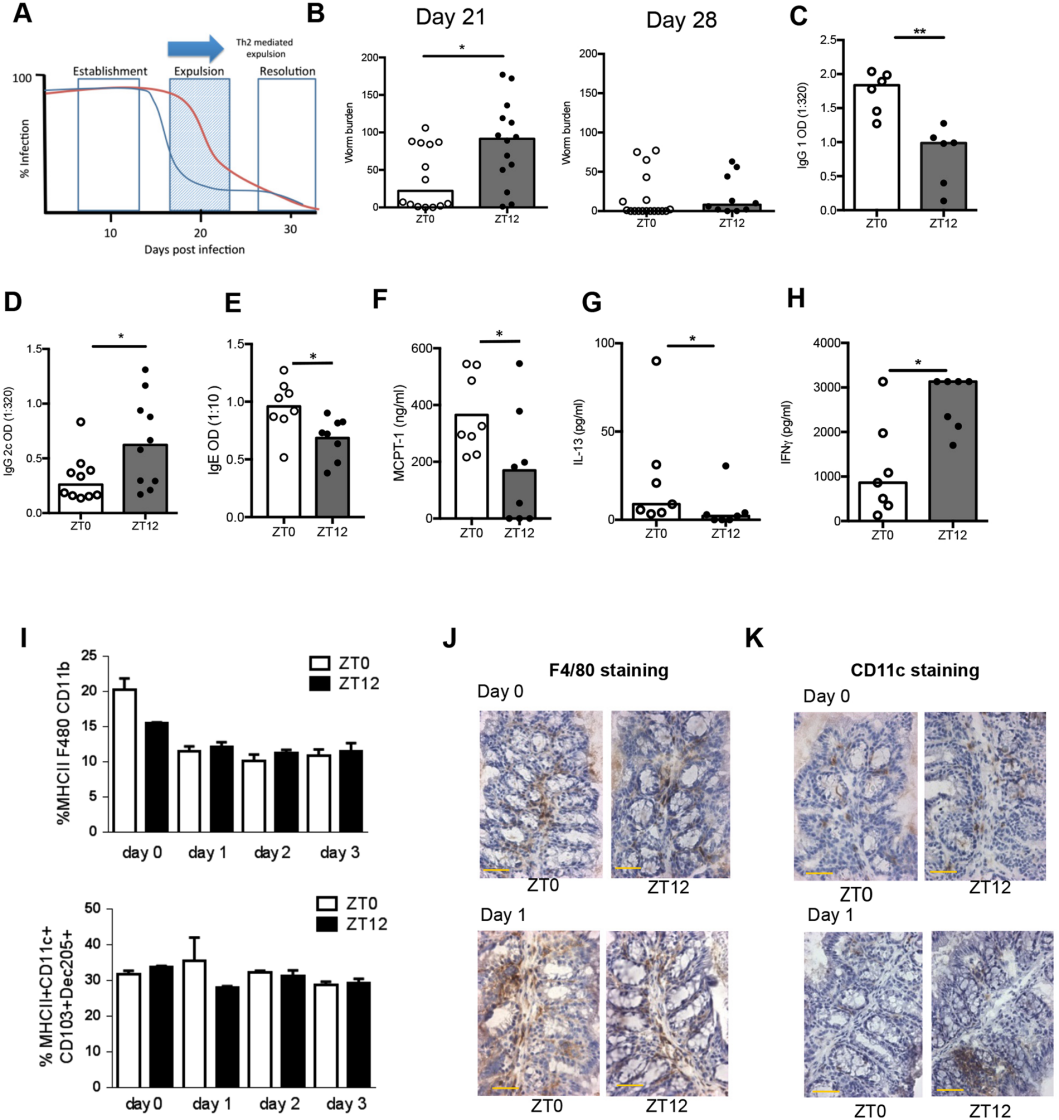
Time of infection with *Trichuris muris* affects kinetics of expulsion. (**A**) The kinetics of expulsion of *T*. *muris* from the C57BL/6 mouse host. Worms establish in all mouse strains prior to expulsion (Establishment). The normal time course of expulsion, mediated by a Th2 response, is shown in blue and delayed expulsion by the red line (Expulsion). In C57 strains, expulsion is complete by approximately Day 30 (Resolution). (**B**) C57BL/6 mice were infected with 200 *T*. *muris* eggs at ZT0 or ZT12, and sacrificed at day 21 or 28, and worm burden accessed in the colon and caecum, combined data from 2 independent experiments, Mann Whitney tests. (**C**,**D**) Parasite-specific IgG1 (n = 6) and IgG2c (n = 10) production on days 21 and 28 respectively. Serum was serially diluted and screened against parasite ES antigen (0.5 μg/ml); data shown is 1:320 dilution, Mann Whitney tests. (**E**) Total IgE (day 21; n = 8 per group). (**F**) Mucosal mast cell protease-1 (MCPT-1; day 21; n = 8 per group) Mann Whitney tests. Mesenteric lymph node cell (MLN) IL-13 (**G**) and IFNγ (**H**) profiles at day 21post infection either at ZT0 or ZT12 cultured with *T*. *muris* E/S (50 µg/ml) for 48 h. Supernatants analysed using cytometric bead array (CBA), n = 8. Maximum level of detection for IFNγ 3000 pg/ml. Mann Whitney tests; data is representative of 2 repeats. All bars represent median. (**I**) Quantification of macrophages and dendritic cells in the large intestinal lamina propria by flow cytometry 0–3 days post infection, n = 3; Kruskal-Wallis test. Immunohistochemical staining for macrophages (**J**) (F4/80) and dendritic cells (**K**) (CD11c) in gut tissue on the day of or 1 day post infection. Positive cells are stained with DAB (Brown), hameatoxylin (blue) counterstain, scale bar represents 100 μm. See also Fig. S1.

Different inbred strains of mice vary in their ability to expel a *T*. *muris* infection^32^ with some strains expelling the parasite within three weeks of infection, while others require more than four weeks, and yet further strains are completely unable to clear the infection. We used the C57BL/6 mouse strain which mounts an immune response characterised by both T_H_1 and T_H_2 cytokines, typically leading to expulsion of parasites approximately three to four weeks later. As in all mouse strains, C57BL/6 mice become infected by the parasite with no expulsion before day 13 post infection (Schematic Fig. 1A; Establishment). Subsequently the developing adaptive immune response, characterised by both T_H_1 and T_H_2 cytokines, leads to expulsion of parasites approximately three to four weeks later (Resolution). If C57BL/6 mice become more biased towards a T_H_1 immune response, this results in delayed-expulsion kinetics, with an elevated worm burden, as indicated by the red line, Schematic Fig. 1A; Expulsion).

The impact of time-of-day of infection on long-term parasite expulsion dynamics was measured using a dose of 200 *T*. *muris* eggs. Mice were maintained on a 12 L:12 D light-dark cycle, and eggs administered via oral gavage at lights on (ZT0; Zeitgeber Time 0) or 12 h later at lights off (ZT12). Animals were sacrificed at Day 21 or 28 post-infection, when mice would normally have started to expel or almost completely expelled their worm burden respectively^32,33^. Mice infected at ZT12 had a significantly higher *T*. *muris* worm burden at Day 21, but both groups had largely cleared worms by Day 28 (Fig. 1B). Infection of any mouse strain with *T*. *muris* eggs results in the establishment of larval stage parasites around two weeks post infection, with this infection subsequently expelled if a dominant T_H_2 immune response is generated^32,34^. It is possible therefore, that the differences in expulsion kinetics observed were simply attributable to time-of-day differences in the initial extent of establishment of infection. To assess this, we quantified worm numbers in mice infected at Day 13, a time-point prior to the normal onset of expulsion. We observed no significant differences between ZT0 and ZT12 infected mice (Fig. S1A). Thus collectively this suggests that long-term programming of the adaptive immune response to parasitic worm infection is determined by the time-of-day of infection, but with manifestation of the effect occurring several weeks after infection.

IgG1 and IL-13, typical markers of the T_H_2 response associated with effective worm clearance, were significantly higher following a ZT0 infection (Fig. 1C,G). Total IgE and mucosal mast cell protease-1 (MCPT-1), which also correlate with the T_H_2 response associated with Trichuris infection were significantly elevated in ZT0-infected mice (Fig. 1E,F). In contrast, IgG2c and IFN-γ, markers of a worm-susceptible T_H_1 immune response, were significantly raised following a ZT12 infection (Fig. 1D,H). Here, IgG2c was measured at day 28 post-infection as the optimal time point post-infection to detect differences in this antibody isotype, At day 21 post infection parasite specific IgG2c levels are usually too low in sera to use as a surrogate marker of delayed expulsion. In subsequent experiments we focussed our final autopsy on day 21 in order to monitor the delayed worm expulsion phenotype as our main readout of clock regulation. Leukocyte populations infiltrate the intestine after a *T*. *muris* infection, with DCs then migrating to the local draining lymph nodes where they initiate T cell polarisation^35^. To assess the early leukocyte recruitment to the gut we quantified two key sentinel cells, the macrophage (MHC II^+^, F4/80^+^ CD11b^+^) and the DC (MHCII^+^, CD11c^+^, CD103^+^ Dec205^+^), in the large intestinal lamina propria by flow cytometry (Fig. 1I, on Days 0, 1, 2 and 3 post infection) and immunohistochemistry (Fig. 1J,K, on Day 0 and Day 1 post infection). These data revealed no significant differences in numbers, which might underlie the observed time-of-day of infection phenotype despite a non-significant trend for fewer macrophages in the lamina propria prior to infection at ZT12. Therefore, the early local intestinal immune response to post worm infection was not affected by time of exposure.

To establish the range of time-of-day influences over worm expulsion we further infected mice at the two intermediate time points (ZT6 and ZT18; Fig. S1B) and assessed worm burdens at day 21 post-infection. These did not differ significantly suggesting that the difference in time-of-day of worm expulsion is at its greatest when comparing ZT0 to ZT12. Analyses of antibodies and cytokines revealed a significant increase in IgG1 after a ZT6 infection and a significantly elevated IFNγ response after a ZT18 infection (Fig. S1C,F) suggesting that the circadian influence over the immune response is still apparent at these two circadian times, but insufficiently polarised to result in a delayed worm expulsion. Thus all subsequent analyses focussed on ZT0 and ZT12. Collectively these data reveal a powerful effect of time-of-day of infection on the kinetics of worm expulsion, with the maximal effect observed at the transitions between light and dark.

### The role of metabolic cues and DC subsets in parasite expulsion

**Figure 2:**
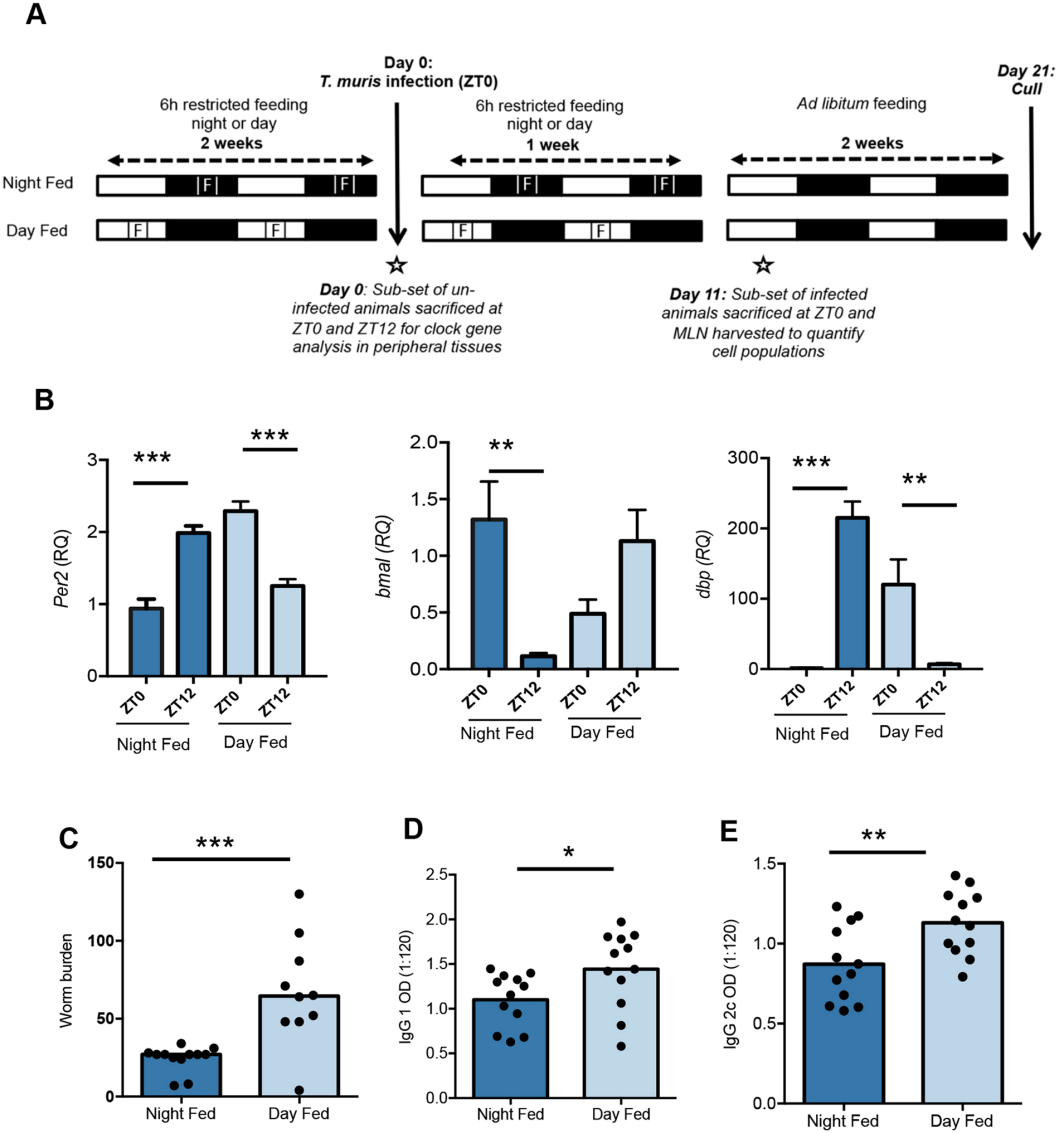
Restricting feeding to the day reverses the gut clock and impairs worm expulsion. (**A**) Schematic of the experimental design - food availability (marked F) was restricted to a 6 h window either mid-day (Day Fed) or mid-night (Night Fed). This restriction was in place 14 days prior to and 7 days into *T*. *muris* infection. Both groups of animals were infected at ZT0 and culled 21 days post infection. (**B**) After 14 days of restricted feeding a cohort of animals were sacrificed at ZT0 or ZT12 to assess rhythms in clock gene expression in peripheral tissues (gut) via QPCR, n = 6/group, Two way ANOVA and post hoc Tukey. (**C**) Worm burden was assessed in gut 21 days post infection, value presented is median n = 10–12, Mann Whitney test. Parasite specific IgG1 (**D**) and IgG2c (**E**) production on day 21, n = 12, data shown is dilution 1:120. Values shown are means, unpaired T test. See also Figs S2 and S3.

We next assessed whether metabolic feeding-associated cues might be involved in directing circadian regulation of worm expulsion. Feeding cues are now widely recognised as a powerful Zeitgeber for peripheral oscillators^36^. To address this we adopted a restricted feeding regime (RFR^37^;), whereby mice (which usually feed mainly at night) have restricted access to food in a 6 h window centred either at mid-light or mid-dark phase. This RFR was imposed for two weeks prior to, and one week post-infection at ZT0 (Fig. 2A). Analysis of core circadian genes (*per2*, *bmal1*, *dbp*) in the intestine at the time of infection revealed that restricting feeding to the daytime reversed the pattern of expression of local clock genes (Fig. 2B). Analysis of the same circadian genes in the liver demonstrated similar outcomes (Fig. S2).

Mice restricted to feeding in the daytime two weeks prior to a ZT0 infection harboured significantly more worms at Day 21 post-infection compared to night-fed mice (Fig. 2C). Surprisingly unlike the time-of-day infection experiments, the effect of food reversal on expulsion kinetics was not associated with alteration in the IgG1/IgG2c balance, with the IgG1 antibody isotype raised following daytime feeding in addition to IgG2c (Fig. 2D,E). The expression of T_H_2 (*il-13*, *Retnlb*, *ccl2*)-associated genes in the local intestinal tissue likewise was elevated following daytime feeding (Fig. S3). The data thus suggests that a transient period of food reversal prior to and early in the infection process is sufficient to regulate the circadian machinery in the gut to impair worm expulsion even after returning to *ad libitum* feeding, but without perturbing the T_H_ balance.

**Figure 3:**
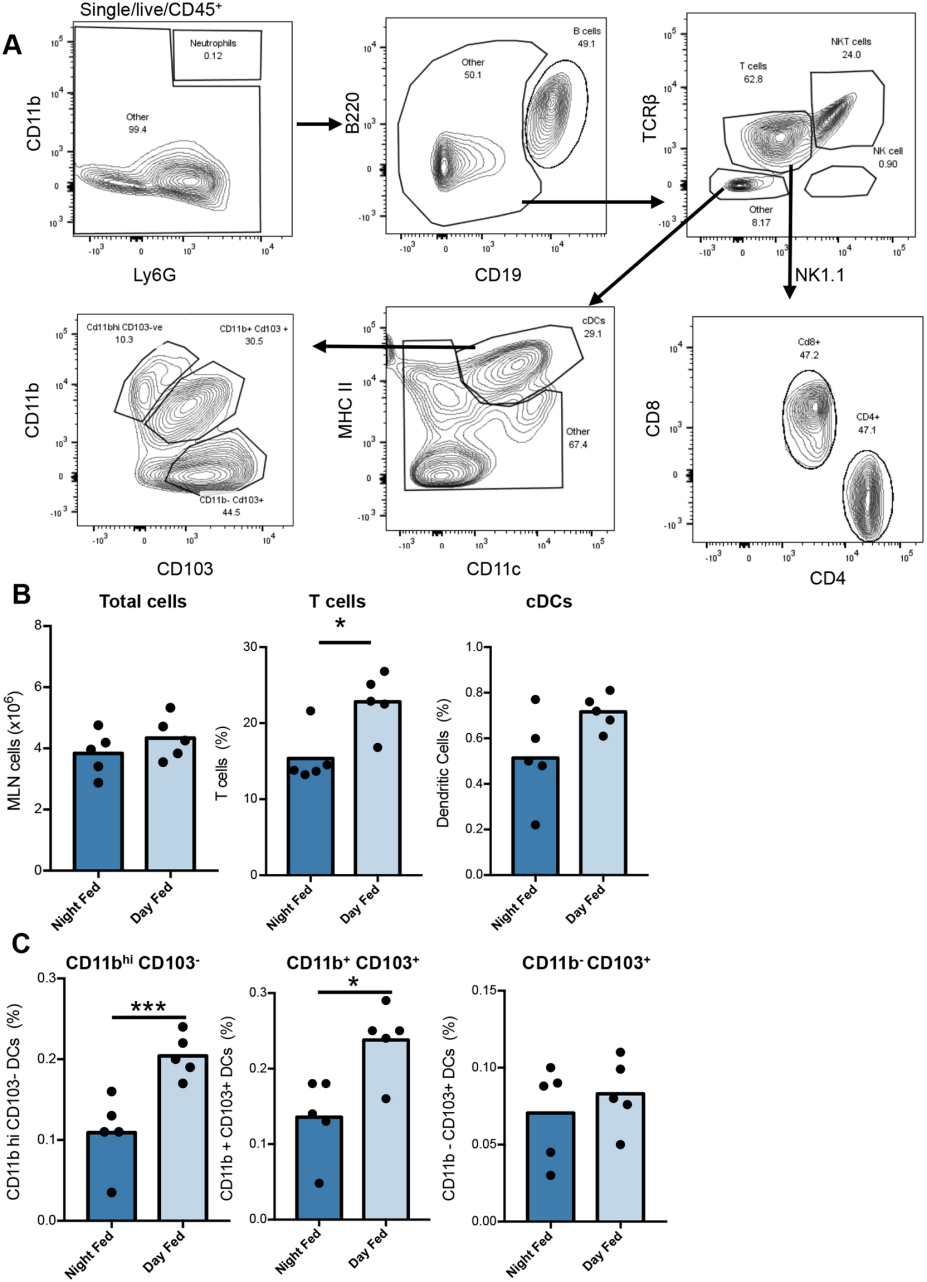
Consequence of restricted feeding regimes on cell populations of the MLN. (**A**) Flow cytometric gating strategy for quantifying individual cell populations in the MLN of mice 11 days post *T*. *muris* infection at ZT0 following restricted feeding regime. (**B**) Quantification of select populations of MLN cells. (**C**) Quantification of select populations of cDC values shown are mean, n = 5/group, unpaired T-tests. See also Table S1.

Infection occurs on ingestion of embryonated parasite eggs, which hatch approximately 90 minutes later upon reaching the caecum^38^. Hatching releases the first stage larva (L1), which burrows into intestinal epithelial cells, and triggers the immune system^35^. Antigen-bearing DCs leave the gut mucosa via the lymphatics, arriving in the mesenteric lymph nodes (MLN) and promote T cell activation from approximately Day 7 post-infection^35^. Thus we investigated the cellular composition of the MLN at Day 11 post-infection, (a time point prior to the onset of worm expulsion) in the more susceptible day-fed versus the more resistant night-fed animals. Flow cytometric analysis (Fig. 3A**)** revealed no significant differences in total cell numbers or in the relative percentages of B cells, NK cells, neutrophils and conventional dendritic cells (cDC) between day-fed and night-fed mice (Fig. 3B and Table S1). However the relative percentage of T cells (both CD4^+^ and CD8^+^) was significantly elevated in day-fed animals (Fig. 3B, Table S1).

In order to investigate changes in cDC subsets, total cDCs were broken down into CD11b^hi^/CD103^−^, CD11b^+^/CD103^+^ and CD11b^−^/CD103^+^ populations. This separation revealed significant increases in the CD11b^hi^/CD103^−^ and CD11b^+^/CD103^+^ populations in day-fed mice (Fig. 3C). Overall, these data suggest that the gut circadian machinery influences the cellular composition of the mesenteric lymph nodes, in particular the proportions of DC subtypes.

### Chronic infection influences body temperature and activity rhythms, but independently of time of infection

**Figure 4:**
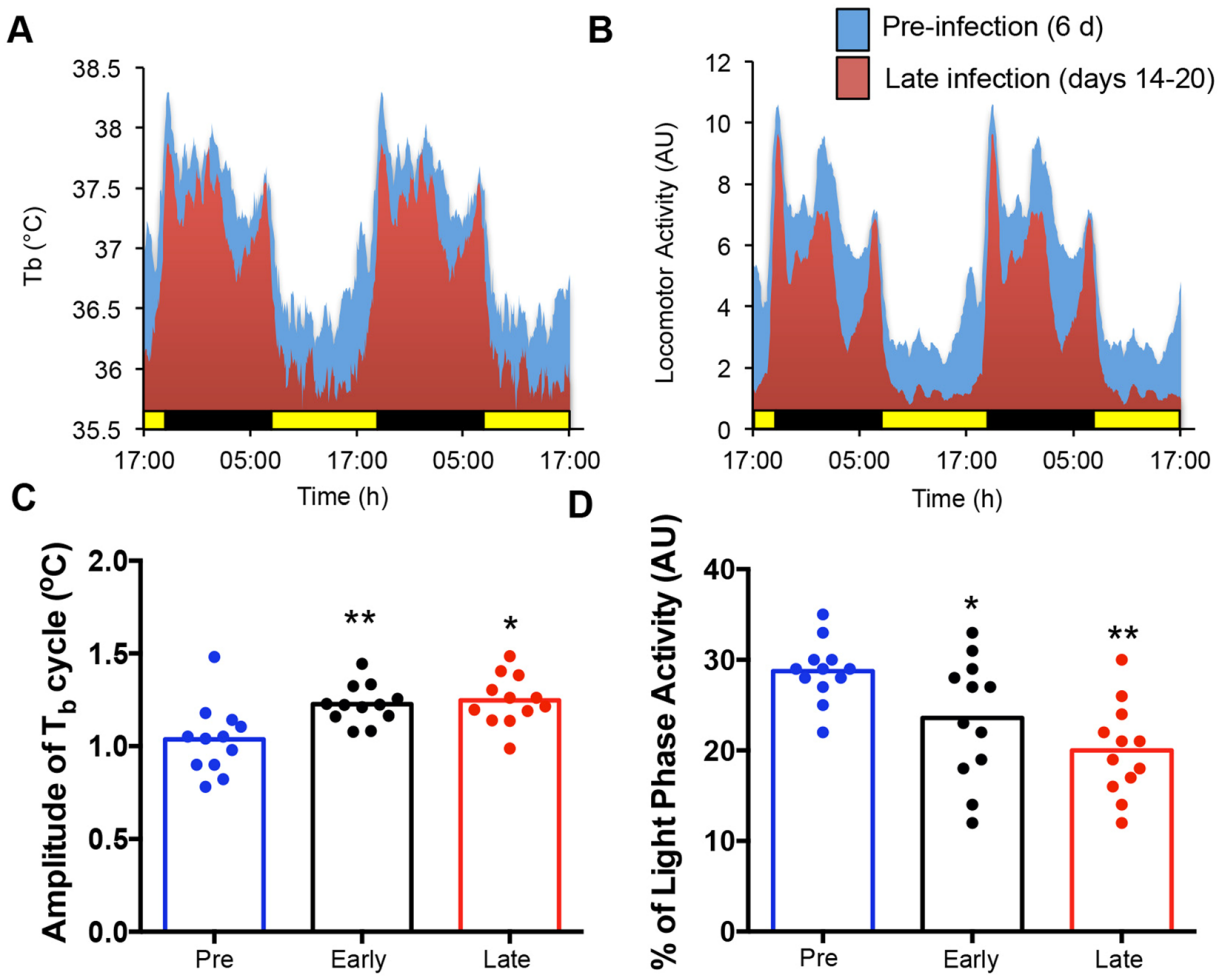
Effect of chronic infection on daily body temperature and activity rhythms. C57BL/6 mice implanted with telemetry devices 3 days prior to infection with 200 *T*. *muris* eggs by oral gavage at ZT0 or ZT12, n = 6/time point. (**A**) Body temperature was recorded in each animal for 6 days prior to infection (Pre - blue), 6 days immediately after infection (Early - black) and for the last 7 days of the study (Late - red). Mice were sacrificed on day 21. Traces shown are the mean body temperature of all animals in the treatment group (**A**,**C**). *T*. *muris* infection caused a significant increase in the daily amplitude of body temperature (averaged for the period of the recordings) (One Way ANOVA and post Hoc Tukey) which was consistent with a significant decrease in basal daytime temperature. Data from mice infected at ZT0 and ZT12 were pooled, as there was no significant effect of infection time on this parameter. (**B**,**D**) Locomotor activity was recorded in each animal; traces shown are the mean activity of all animals in the treatment group. Quantification of daytime activity (pooling ZT0 and ZT12 infected animals) showed decreased activity with *T*. *muris* infection as measured by % of the light phase active (One Way ANOVA and post Hoc Tukey). See also Fig. S4.

Parasitic infection can lead to widespread systemic disruption of circadian-regulated physiology^39,40^. To assess whether infection with *T*. *muris* leads to a widespread systemic perturbation of circadian organisation and behaviour we used whole body telemetry. Mice display strong diurnal oscillations in both body temperature and activity, which continued following infection (Fig. 4A,B). Comparison of mice infected at ZT0 and ZT12 showed no significant difference in daytime body temperature or activity (Fig. S4). However, a significant increase in the mean amplitude of body temperature was recorded after infection that was independent of time-of-day of infection (Fig. 4C). Infected mice showed a significant reduction in overall activity, likely a sickness response (Fig. 4D, Fig. S4). Further, mice in light-dark cycles typically exhibit a short period of “anticipatory behaviour” to dark-onset with an increase in activity in late light phase, but by late stages of infection this did not occur (Fig. 4B). These data indicate that although infection slightly altered the phasing of activity onsets, robust rhythmic behaviour was maintained and was not significantly modified by infection.

### DCs drive circadian responses

**Figure 5:**
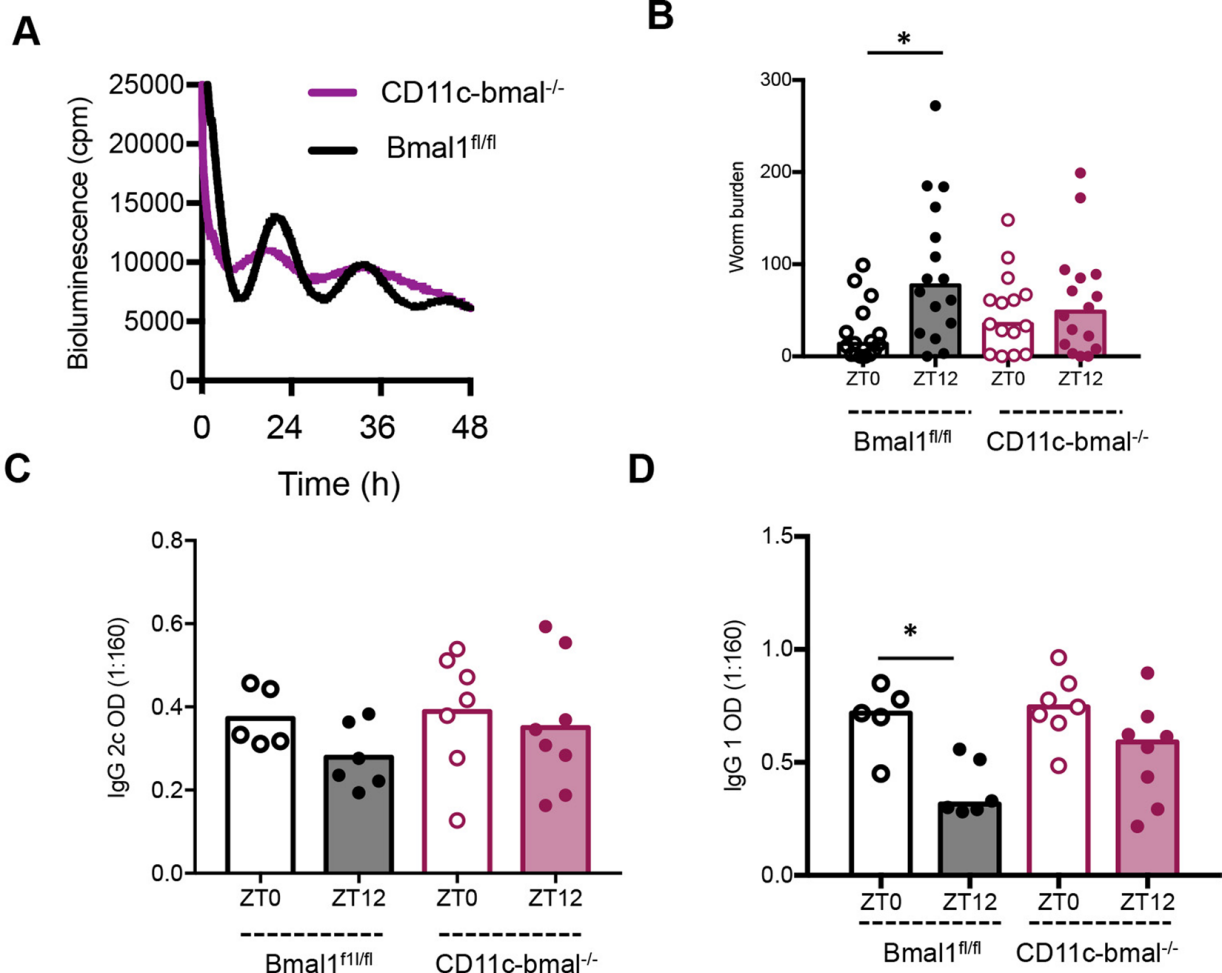
Conditional deletion of *bmal1* in dendritic cells abolishes diurnal variation in immune response to infection. Mice were generated which lacked *bmal1* in CD11c positive cells (CD11c-bmal^−/−^). (**A**) Bone marrow derived dendritic cells cultured from CD11c-bmal^−/−^ mice on a PER2::luc background and their wildtype counterparts (bmal^fl/fl^), were placed under photonmultiplier tubes (PMT) to monitor real-time luciferase activity as a readout of PER2 expression. (**B**) CD11c-bmal^−/−^ and bmal^fl/fl^ littermates were infected with *T*. *muris* at either ZT0 or ZT12. Worm burden (median presented) was assessed 21 days post infection, n = 15–16/group, combined data from two independent experiments. One way ANOVA, post hoc Tukey. (**C**,**D**) Parasite specific IgG2c and IgG1 production was measured on day 21. Serum was serially diluted and screened against parasite ES antigen (0.5 μg/ml); the data shown is dilution 1:160 only, as it falls within the linear range of the titration curve, n = 5–8, One Way ANOVA and post-hoc Tukey.

We next addressed how the DC circadian clock may be involved, by deleting the core circadian clock gene *Bmal1*. Mice homozygous for a floxed *Bmal1* allele (*Bmal1*^*fl/fl*^) were bred with mice expressing Cre-recombinase driven by a CD11c transgene, which is expressed in DCs (CD11c*-Bmal1*^−/−^). All mice were maintained on a background strain carrying the PER2::Luciferase circadian reporter construct^41^. Cultured bone marrow derived DCs from *Bmal1*^*fl/f*l^ mice exhibited robust PER2 bioluminescent circadian oscillations (Fig. 5A). Strikingly, DCs derived from CD11c*-Bmal1*^−/−^ mice showed significantly dampened oscillations. Next, we infected CD11c-*Bmal1*^−/−^ and “floxed” (*Bmal*^fl/fl^) littermates with *T*. *muris* at either ZT0 or ZT12. As predicted from earlier studies, ZT12-infected *Bmal1*^*fl/fl*^ mice harboured significantly more worms at Day 21 post-infection compared to ZT0 (Fig. 5B). In contrast, worm burdens of ZT0 and ZT12-infected, CD11c-*Bmal1*^−/−^ mice were not significantly different, suggesting a loss of circadian control over expulsion kinetics (Fig. 5B). Levels of the T_H_1 marker IgG2c were low in all groups of mice at Day 21 post-infection and showed no significant differences between groups (Fig. 5C). However, in keeping with the increased worm burden in ZT12-infected *Bmal1*^*fl/fl*^ mice the signature T_H_2 marker IgG1 was significantly reduced only in *Bmal1*^*fl/fl*^ mice infected at ZT12, all other groups exhibited IgG1 levels typically associated with a more efficient worm expulsion (Fig. 5D). Thus, these data suggest that *Bmal1* plays a crucial role in the regulation of resistance to *T*. muris infection, and that loss of *Bmal1* in DCs results in loss of the time-of-day variation in worm expulsion, favouring development of a protective immune response across the day.

### The loss of *bmal1*^−/−^ in DCs reduces expression of T_H_1 promoting cytokines

**Figure 6:**
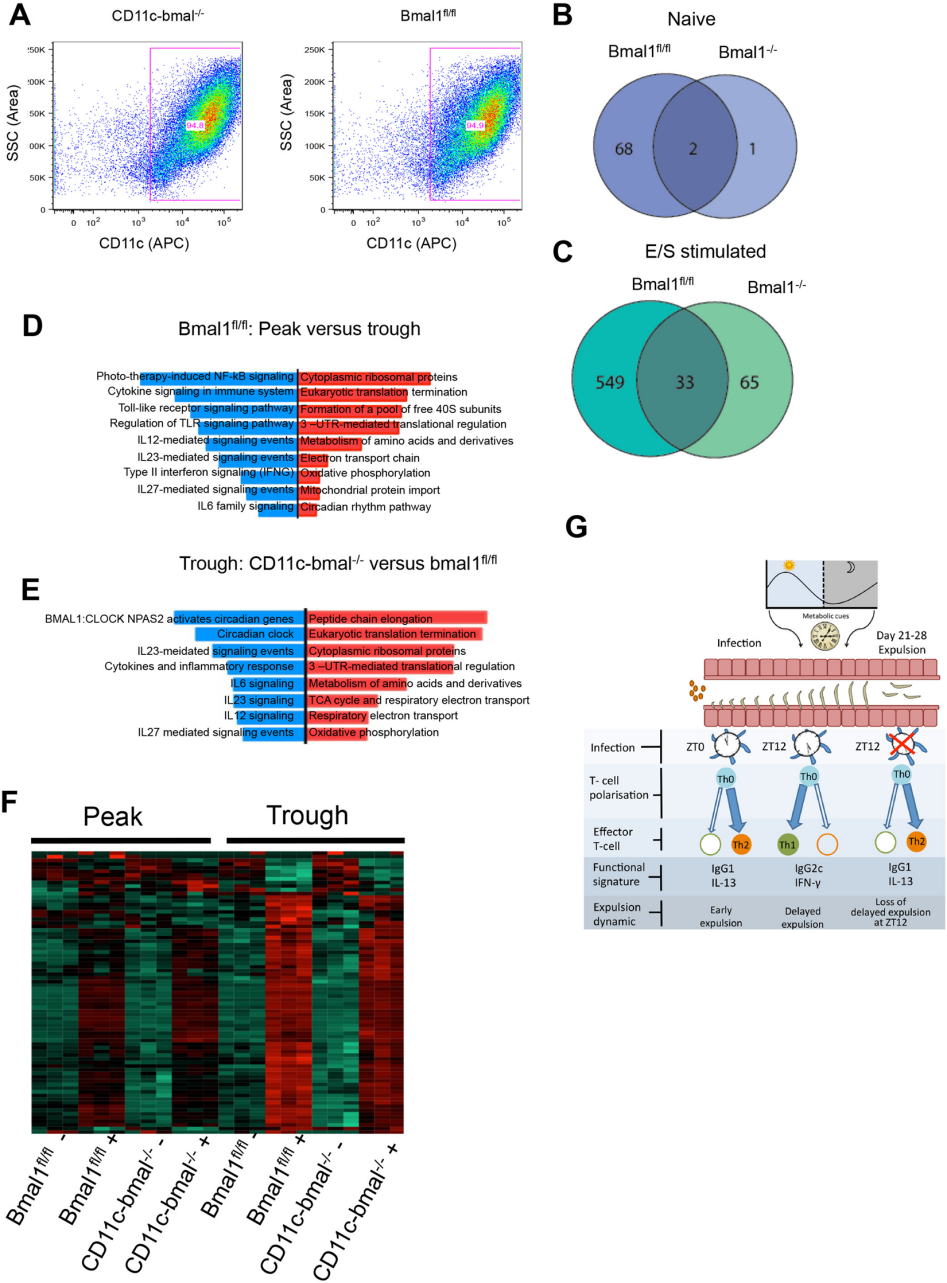
The effect of *bmal1* deletion in dendritic cells on circadian-gated responses to ES antigen. Bone marrow derived DCs were cultured from Bmal^fl/fl^ and CD11c-bmal^−/−^ mice and (**A**) purity confirmed by flow cytometry. After 48 h temperature synchronisation, cells were challenged for 3 h with ES antigen (or vehicle) at either the peak or trough of PER2::luc activity and RNA-Seq was performed. Data represents 3 technical repeats. Venn diagrams showing genes exhibiting significant (Padj < 0.05) diurnal variation in the two genotypes in naïve cells (**B**, blue) and ES stimulated cells (**C**, green). (**D**) Pathway analysis mapping differences in gene expression between Peak and Trough ES challenge in bmal^fl/fl^ mice. Blue bars, pathways down-regulated at peak, red bars, pathways up-regulated at peak. (**E**) Pathway analysis mapping differences between CD11c-bmal^−/−^ and bmal^fl/fl^ cells after stimulation with ES antigen at the trough of PER2::luc activity. Blue bars, pathways down regulated in CD11c-bmal^−/−^. Red bars, pathways up-regulated in CD11c-bmal^−/−^. (**F**) Heat map showing gene expression across the treatment groups, highlighting increased induction of genes after ES stimulation in bmal^fl/fl^ versus CD11c-bmal^−/−^ mice at the trough of PER2::luc activity. (**G**) Schematic illustrating the role of time-of-day of infection on parasitic worm expulsion. See also Fig. S5 and Tables S2 and S3.

To investigate the role of *Bmal1* in DCs, gene expression profiles were compared using bone marrow derived DCs as a surrogate for the tissue DC. Bone marrow derived DCs from *Bmal1*^fl/fl^ and CD11c-*Bmal1*^−/−^ mice were isolated and cultured to high purity (>94%; Fig. 6A). Following synchronisation with temperature cycles^42^ cells were treated with *T*. *muris* ES antigen or vehicle at either the peak or trough of PER2::luc bioluminescence activity in *Bmal*^fl/fl^ PER2::luc cells. Identical time-points were used for the non-cycling CD11c-*Bmal1*^−/−^ PER2::luc derived DCs which exhibited markedly dampened bioluminescence rhythms, as shown previously (Fig. 5A). Cells were collected for RNA-SEQ analysis 3 h post antigen challenge.

In unchallenged (naïve) *Bmal*^fl/fl^ DCs 70 genes were significantly different between peak and trough. Whereas in *Bmal1*^−/−^ DCs only 3 genes exhibited significant differences between peak and trough (Fig. 6B). In ES-stimulated DCs we saw an increase in the number of genes differing at peak versus trough in the *Bmal*^fl/fl^ and bmal1^−/−^ DCs. However, the number of genes differing in the *Bmal*^fl/fl^ DC was considerably higher (Fig. 6C). Following ES-stimulation we saw differences between peak and trough in *Bmal1*^*fl*^/^*fl*^ DCs in the expression of genes associated with T_H_1 polarisation (Table S2). This included *il-12b* and *Tnfsf15* which were significantly down-regulated at peak (Table S2). Further, pathway analyses of the differentially expressed genes in ES stimulated *Bmal1*^*fl/fl*^ DCs at peak versus trough revealed major differences in pro-inflammatory and T_H_1 associated pathways. These pathways include the IL-12 and IL-27-mediated signalling pathways, which were down-regulated at peak (Fig. 6D). In addition, changes in metabolic pathways were evident, with increases in, for example, amino acid metabolism and oxidative phosphorylation pathways in *Bmal*^fl/fl^ DC at peak. Using String analysis^43^ we assessed the interconnectivity of genes that differed at peak versus trough in *Bmal*^fl/fl^ DC (Fig. S5A). Within this gene-set significantly more genes were seen to be interacting than would be expected by chance (P < 10^−14^).

There were few differences between *Bmal1*^*fl/fl*^ and *Bmal1*^−/−^ DCs at the peak PER2 bioluminescence following antigen challenge (Fig. 6F). In marked contrast, comparison of *Bmal1*^*fl/fl*^ and *Bmal1*^−/−^ DCs at the trough of PER2 bioluminescence revealed differences (Fig. 6F) with significant down-regulation of T_H_1 genes in the *Bmal1*^−/−^ DCs (Table S3). Both the IL-12 and IL-27 pathways were also down-regulated in cells lacking *Bmal1* (Fig. 6E). In addition, loss of *Bmal*1 from DCs was associated with an up-regulation in metabolic pathways including amino acid metabolism and the TCA cycle. We also assessed the interconnectivity of genes that differed between genotype from cells sampled at the trough of the PER2::Luc cycle by String analysis (Fig. S5A**)**. In this analysis, *Bmal1*-regulated genes emerged as a tightly connected cluster (P < 10^−14^).

Collectively these data from cultured circadian-synchronised DCs stimulated with parasite antigen reveal that T_H_1-associated genes are circadian regulated. Cytokine and signalling pathways associated with the development of T_H_1 immune responses were down-regulated in the absence of *Bmal1*. Thus, the clock regulator BMAL1 may be critical for the establishment of DC-derived T_H_1 promoting cytokines (Fig. 6H).

## Discussion

The circadian clock is recognised as a key regulator of immune cell function, with molecular clock control of inflammation, sepsis and immunity to bacterial and protozoan parasite infection described^15,26,31,44^. More recently, remarkable time-of-day differences in lymphocyte trafficking and adaptive immune responses have emerged^29,30^. However, the role of the circadian clock in regulation of immunological responses to large multi-cellular parasites, that are eliminated by T_H_2 immune responses, remain unexplored. To address this question, we took advantage of a gastro-intestinal nematode parasite infection model where expulsion of the pathogen is exquisitely dependent on the T_H_1/T_H_2 cell balance, thus enabling changes in this balance to be evidenced by altered worm expulsion kinetics and the balance of parasite-specific IgG1/IgG2c.

We now show that time of infection is a key component in tuning the efficiency of worm expulsion. Our data show that mice infected in the early light-phase were more efficient in expelling their worm burden than mice infected at the onset of the dark-phase, with cytokine signatures (increased IL-13, reduced IFN-γ), and parasite-specific antibody profiles (increased IgG1; reduced IgG2c) consistent with an immune response polarised towards T_H_2. These effects were not immediate, but become manifest over several weeks, and were not associated with the altered worm establishment. Food reversal studies also strongly implicated an important role for metabolic cues during the initial stages of infection. Thus inversion of the local gut clock, by restricting feeding to the daytime, impaired parasitic immune responses. Interestingly, unlike time-of–day of infection, food reversal impaired worm expulsion in the presence of T_H_2 immune responses. This was accompanied by increases in CD11b^hi^/CD103^−^ and CD11b^+^/CD103^+^ cDC with CD11b^+^ cDC thought to be the main drivers of T_H_2 immune responses *in vivo*^45^. One possible implication of this is that reversed-feeding leads to a state of internal de-synchrony between central and peripheral signals^46^. Thus, central signals (T_H_2) defining efficient worm expulsion after a ZT0 infection may be unable to coordinate the innate T_H_2-controlled effector mechanisms in intestinal tissue subjected to a reversed feeding regime. To our knowledge, this represents the first demonstration that cellular immune responses underlying worm expulsion are sensitive, either directly or indirectly, to feeding-associated metabolic cues. Interestingly, in keeping, tissue innate lymphoid cells (ILC) 2s have previously been shown to influence tissue eosinophilia via a mechanism sensitive to nutrient intake^47^. The alterations in the relative percentages of DC subsets and T-cells in the MLN observed when feeding was restricted to day-time are in keeping with the recent reports^29,30^ showing strong diurnal effects on leukocyte migration and concurrent accumulation of T cells at night compared to day under steady state conditions. Our data from RFR studies, suggests, at least for the mesenteric lymph nodes (MLN), metabolic cues may contribute to this effect. We currently do not know if the RFR regime has ablated oscillations or shifted oscillations and this warrants further investigation. Further, we suggest that populations of cDC within the MLN may also be under circadian control.

Our data identify the DC as a key cell type underpinning the BMAL1 regulated resistance to *T*. *muris in vivo*. Thus, mice with *Bmal1*-deficient DCs no longer showed a time-of-day dependent effect on worm expulsion, with both ZT0 and ZT12-infected mice harbouring equivalent worm burdens to ZT0-infected *Bmal*^fl/fl^ littermate controls. Although our current studies cannot definitively show that the circadian clock regulates resistance to *T*. *muris* infection *in vivo*, we can conclude that loss of BMAL1 in DCs results in loss of the time-of-day variation in worm expulsion. To begin to explore further how BMAL1 in DCs regulates the time-of-day dependency of worm expulsion we used circadian-synchronised purified bone marrow derived DCs stimulated with *T*. *muris* antigen. These data revealed BMAL1 as an important component in determining the ability of the DC to promote polarised T cell responses with a down-regulation in T_H_1-promoting cytokines and pathways in BMAL1-deficient cells. Furthermore, we also show that these differences are both DC-intrinsic and circadian-regulated. This supports a mechanism that involves dynamic changes over the day in the promotion of T_H_1-promoting cytokines (e.g. IL-12) and T_H_1-pathways in non-targeted (*Bmal1*^fl/fl^) DCs, which are circadian-regulated. Interestingly, two of the major signalling pathways down-regulated in BMAL1 deficient DCs were the IL-12 and IL-27-mediated signalling pathways, both known to be critical in enabling T_H_1-mediated immune responses, and failure to expel *T*. *muris*^6,48^. Further studies are warranted to translate our *in vitro* driven hypothesis to *in vivo*, exploring the early post infection cellular events underpinning the time of day effects on worm expulsion which emerge three weeks post infection.

Our study adds the DC to the repertoire of circadian-regulated immune cells in which components of the core molecular clock-work have been targeted, with impact on either innate and/or adaptive responses^18,30,49,50^. We selected *Bmal1* for genetic targeting, as uniquely loss of this transcription factor leads to suppression of rhythmic activity of the core mammalian clockwork^51,52^. Given that *Bmal1* is the critical regulator of the other clock genes which comprise the molecular clock, it remains to be defined whether the effects of *Bmal1* deficiency are through effects on downstream clock proteins, such as REV-ERB or CRYPTOCHROME, or are directly BMAL1-regulated.

In summary, we show that expulsion of *T*. *muris* is influenced by time-of-day of infection and involves entrainment by metabolic feeding cues. BMAL1 and clock-control of DC function now emerge as important components in the efficient expulsion of helminth parasites.

## Methods

### Mice

All experimental procedures and protocols were carried out in accordance with the guidelines of the animals (Scientific Procedures) Act, 1986 and subject to local ethical approval by the University of Manchester Animal Welfare and Ethical Review Board. Male C57BL/6J mice (6–8 weeks) were purchased from Harlan. Unless otherwise stated, mice were maintained at a temperature of 20–22 °C in a 12 h light, 12 h dark lightening schedule, in sterile, individually ventilated cages with food and water *ad lib*. A CD11c^cre^ line^53^ was bred with a bmal1^fl/fl^PER2::luc line^18^ to generate CD11c-bmal^−/−^ mice and Bmal1^fl/fl^, on a C57BL/6J background, which were used as littermate controls. Radiotelemetry probes (Data Sciences International) were implanted in the peritoneal cavity under isoflurane-induced anaesthesia 3 days prior to infection. During the restricted feeding regime (RFR) access to food was provided either mid-light phase (ZT4 – ZT9; Day fed) or mid-dark phase (ZT16 – ZT21; night fed).

### Infection

The parasite was maintained as previously described^38^. 200 eggs were given per oral gavage to each mouse. For worm counts, caecum and colon were harvested, opened and the gut mucosa scraped to remove epithelia prior to screening for *T*. *muris* worms.

### Culture of bone marrow derived dendritic cells

Bone marrow cells were seeded on Day 0 at 2 × 10^5^/ml on 10 cm dishes in RPMI (2% Pen Strep, 10% fetal calf serum and 1% L-glutamine) containing 20 ng/ml GM-CSF. Fresh media containing 20 ng/ml GM-CSF was added on day 3, day 6 and day 8, and cells used on day 10. For photonmultiplier tube (PMT) experiments, DCs were plated on 36 mm dishes in a recording media containing luciferin^54^ synchronised using two full temperature cycles of 12 h 35 °C/12 h 37 °C and placed under the PMTs.

### Mesenteric lymph node (MLN) cell re-stimulation and cytokine bead array

MLN cells were brought to cell suspension and 1 × 10^6^ cells were cultured for 48 h at 37 °C, 5% CO_2_, with E/S antigen (50 µg/ml). Supernatants were analysed by Cytometric Bead Array (CBA) Mouse/Rat soluble protein flex set system (BD Bioscience, Oxford, UK). Results were measured by Flow cytometry. For analysis, FCAP Array v1.0.1 software (BD Cytometric Bead Array) was used.

### Immunohistochemistry

Immunohistochemistry was performed on frozen colon sections. CD11c (clone N418) antibody (eBioscience, Hatfield, UK) was used to identify dendritic cells, F4/80 (clone Cl-A3-1, AbD Serotec) antibodies were used to identify macrophages. Slides were counterstained using Mayer´s haematoxylin (Sigma Aldrich Company Ltd, Dorset, UK).

### IgG ELISA

Serum was assayed for parasite specific IgG1 and IgG2c. 96 well plates were coated with 5 μg/ml T. muris E/S antigen overnight, incubated with serum (2 fold dilutions, 1:20–1:2560). Parasite specific antibody was measured using biotinylated IgG1 or IgG2c (BD Biosciences). The plates were read at 405 nm, with reference of 490 nm.

### IgE ELISA

Serum was assayed for total IgE antibody production. 96 well plates were coated with purified anti-mouse IgE (2 ug/ml, Biolegend, Clone: RME-1) in 0.05 M carbonate/bicarbonate buffer and incubated overnight at 4 °C. Following coating, plates were washed in PBS-Tween and non-specific binding blocked with 3% BSA (Sigma-Aldrich) in PBS for 1 hour at room temperature. Plates were washed and diluted serum (1:10) added to the plate and incubated for 2 h at 37 °C. After washing HRP conjugated goat anti-mouse IgE (1 ug/ml; BioRad) was added to the plates for 1 h. Finally, plates were washed and developed with TMB substrate kit (BD Biosciences, Oxford, UK) according to the manufacturer’s instructions. The reaction was stopped using 0.18 M H_2_SO_4_, when sufficient colour had developed. The plates were read by a MRX II microplate reader (DynexTechnologies, VA, USA) at 450 nm, with reference of 570 nm subtracted.

### MCPT-1 ELISA

Serum MCPT-1 levels were assayed at a 1:100 dilution utilising a mouse MCPT-1 ELISA kit (Invitrogen) according to manufacturer’s instructions.

### Lamina propria lymphocyte (LPL) isolation

Lymphocytes were isolated from the lamina propria of caecum and colon using an enzyme cocktail. containing 1.25 mg/ml collagenase D (Roche, West Sussex, UK), 0.85 mg/ml collagenase V (Sigma-Aldrich Company Ltd; Dorset, UK), 1 mg/ml dispase (Gibco, Life Technologies Ltd; Paisley, UK) and 30 µg/ml DNase (Roche, West Sussex, UK) dissolved in complete RPMI media. 2 × 10^6^ cells were used for FACS staining. FC receptors were blocked using anti CD16/CD32 antibody (1:100, BD Biosciences, Oxford, UK), stained with PE-anti mouse CD103 (clone M290, 1:200, BD Pharmigen, BD Biosciences, Ozford, UK), Alexaflor 700- anti mouse CD45 (clone 30-F11, 1:200, BD Biosciences, Oxford, UK) FITC- anti mouse CD11b (clone M1/70 1:200), PE-Cy7-anti mouse CD11c (clone N418 1:200), Pacific Blue-anti mouse IA-IE (clone M5/114.15.2, 1:400), APC-anti mouse F4/80 (clone BM8,1:200, all eBioscience, Hatfield, UK) and Biotin- anti mouse Dec205 (NLDC-145,1:200 Biolegend, Cambridge, UK) prior to addition of SAv-QDot 605 (Invitrogen, Life Technologies Ltd; Paisley, UK) with Biotin-anti mouse Dec205. 7AAD (10 µL, BD Biosciences, Oxford, UK) was added to 200 µl FACS Buffer remaining in each tube and about 1 × 10^6^ cells were acquired using LSR II Flow cytometer (BD Biosciences, Oxford, UK).

For the analysis of MLN cells 1 × 10^6^ cells were re-suspended in 1:2000 Live/Dead Zombie UV (Biolegend). Fc receptors were then blocked prior to staining with: FITC-anti mouse MHC-II (1:600), PerCP/Cy5.5-anti mouse Ly6G (1:600), PerCP/cy5. 5-anti mouse CD8 (1:600), APC -anti mouse CD4 (1:200), APC-anti mouse CD138 (1:200), AF700-anti mouse Ly6C (1:200), APC/Cy7 -anti mouse TCR-beta (1:200), BV421 -anti mouse CD103 (1:200), BV510-anti mouse XCR1 (1:500), BV605-anti mouse CD11c (1:400), BV650-anti mouse PDCA-1 (1:200) BV711-anti mouse CD11b (1:500), BV785-anti mouse B220 (1:150), PE -anti mouse CD64 (1:100), PE/CF594 -anti mouse CD19 (1:200), PE/Cy5 -anti mouse NK1.1 (1:200) and PE/Cy7 -anti mouse F4/80 (1:200). Cells were washed in FACS buffer and were fixed in 1% PFA (Sigma Aldrich) for 10 minutes are RT before a final wash in FACS buffer. 1 × 10^6^ cells were then acquired on a BD Fortessa. All antibodies were purchased from Biolegend apart from TCRbeta APC-Cy7 (eBioScience) and CD19 PE CF594 (BD).

### qPCR

RNA was extracted after homogenising gut tissue in Trizol using a FastPrep machine with Lysing matrix D (MP Biomedicals). After genomic DNA elimination, 2 μg RNA was converted to cDNA (RNA to cDNA, Applied biosystems). qPCR was performed on a StepOne plus machine using Taqman probes and primers and qPCR MasterMix (Eurogentec). Beta actin was used as a housekeeping gene.

### RNA-Seq analysis

Strand-specific RNA-Seq libraries were prepared using the Illumina workflow. Paired-end reads were generated and 40 to 85 M of total reads obtained from each sample. The fastq files generated by Illumina HiSeq4000 platform were analysed with FastQC and any low quality reads and contaminated barcodes were trimmed with Trimmomatic^55^. All libraries were aligned to GRCm38.p2 (mm10) assembly of mouse genome using Tophat-2.1.0^56^ and only matches with the best score were reported for each read. The mapped reads were counted by genes with HTSeq^57^ against gencode.vM2.annotation.gtf. Differentially expressed (DE) genes were identified by comparing between the treatment groups with DESeq2^58^. The functional analysis was carried out with a R package topGO^59^ and RontoTools.

### Statistics

Where statistics are quoted, Mann-Whitney U test was used for non-parametric data comparing two groups. Kruskal-Wallis test, with Dunn’s multiple comparison post hoc was used to compare three or more groups. Parametric data were assessed using unpaired T tests for pairwise comparisons or one-way ANOVA and post-hoc Tukey. A p-value of < 0.05 was classed as significant, *P < 0.05, **P < 0.01, ***P < 0.005. Statistical analysis was carried out using GraphPad Prism for windows, version 6.

### Data availability

The datasets generated during the current study are available from the corresponding authors on reasonable request.

## Electronic supplementary material

Supplementary Figures
