# Supplementary material for: The circadian regulator BMAL1 programmes responses to parasitic worm infection via a dendritic cell clock
Source: Sci Rep. 2018 Feb 28;8:3782. doi: 10.1038/s41598-018-22021-5 (PMC5830501; doi:10.1038/s41598-018-22021-5)
Supplement: Supplementary file 1 — Supplementary Figures [file 41598_2018_22021_MOESM1_ESM.pdf]

The circadian regulator BMAL1 programmes responses to parasitic worm infection via a dendritic cell clock

Authors: Tom Hopwood[1], Sarah Hall[a1], Nicola Begley[1], Ruth Forman[1], Sheila Brown[2], Ryan Vonslow[1], Ben Saer[1], Matthew Little[1], Emma A Murphy[1], Rebecca Hurst[1], David Ray[1], Andrew MacDonald[2], Andy Brass[1], David Bechtold[1], Julie Elizabeth Gibbs[*1], Andrew S Loudon[*1] and Kathryn J Else[*1]

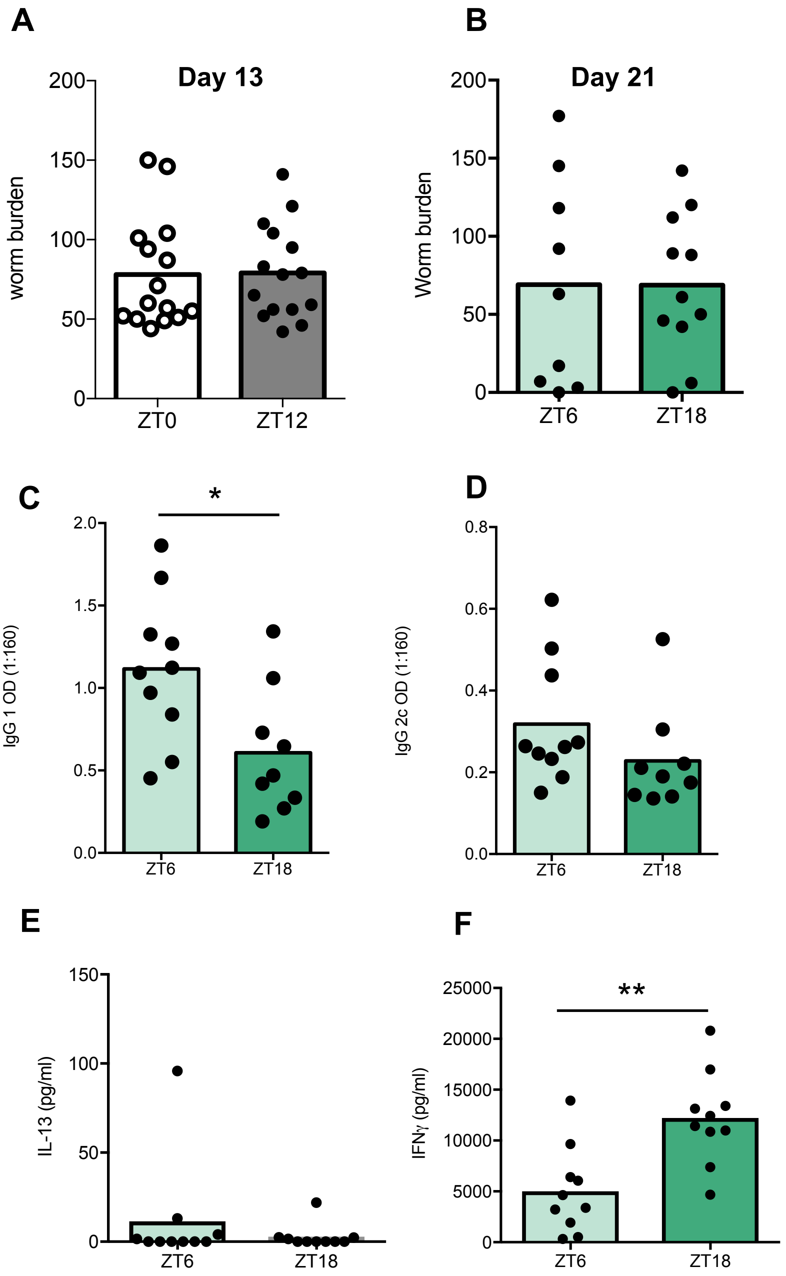

Figure S1: Worm expulsion prior to day 21 and after infection at ZT6 or ZT18. Related to Figure 1.
(A) C57/BL6 mice infected with *T. muris* at ZT0 or ZT12 were culled on day 13 to confirm equivalent worm establishment in the gut, n = 15/ group. (B) C57BL/6 mice were infected with *T. muris* at ZT6 or ZT18 (n=9-11). Mice were sacrificed at day 21, worm burden was accessed by counting worms found in the colon and caecum. (C and D) Parasite specific IgG1 and IgG2c production on day 21 and day 28 respectively, n=9-10. Serum was serially diluted and screened against parasite ES antigen (0.5µg/ml); the data shown is dilution 1/160 only, as it falls within the linear range of the titration curve, unpaired Mann Whitney T test. (E and F) Mesenteric lymph node cell (MLN) IL-13 and IFNγ profiles at day 21 post infection, n=10, unpaired T test.

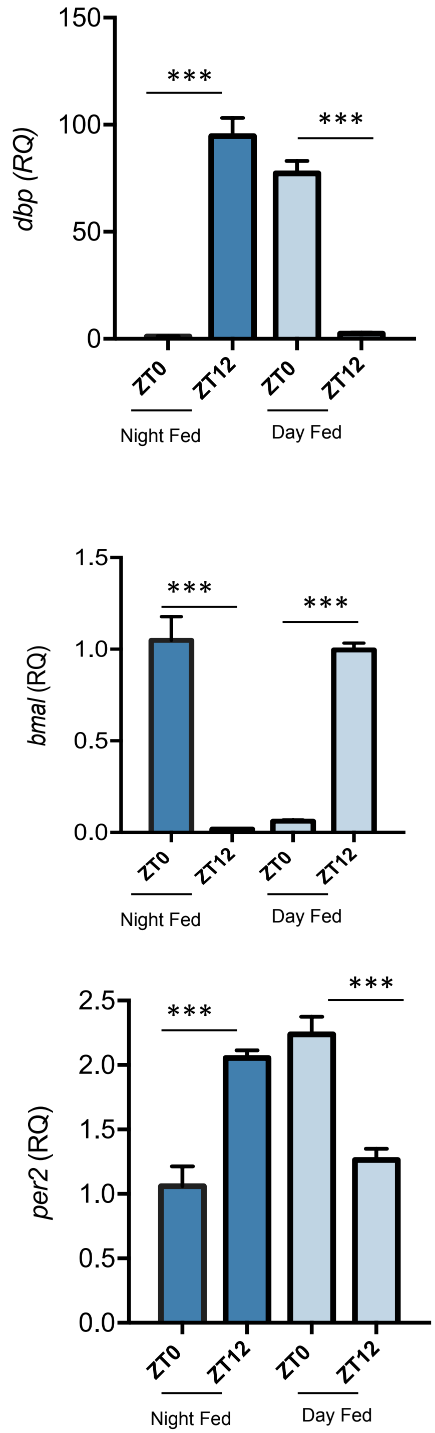

Figure S2: Reversal of the liver clock in response to restricted feeding schedule. Related to Figure 2.
Expression of clock genes in liver tissue harvested from mice (at ZT0 or ZT12) 14 days after initiation of the restricted feeding schedule, n=6/group, Two way ANOVA and post-hoc Tukey.

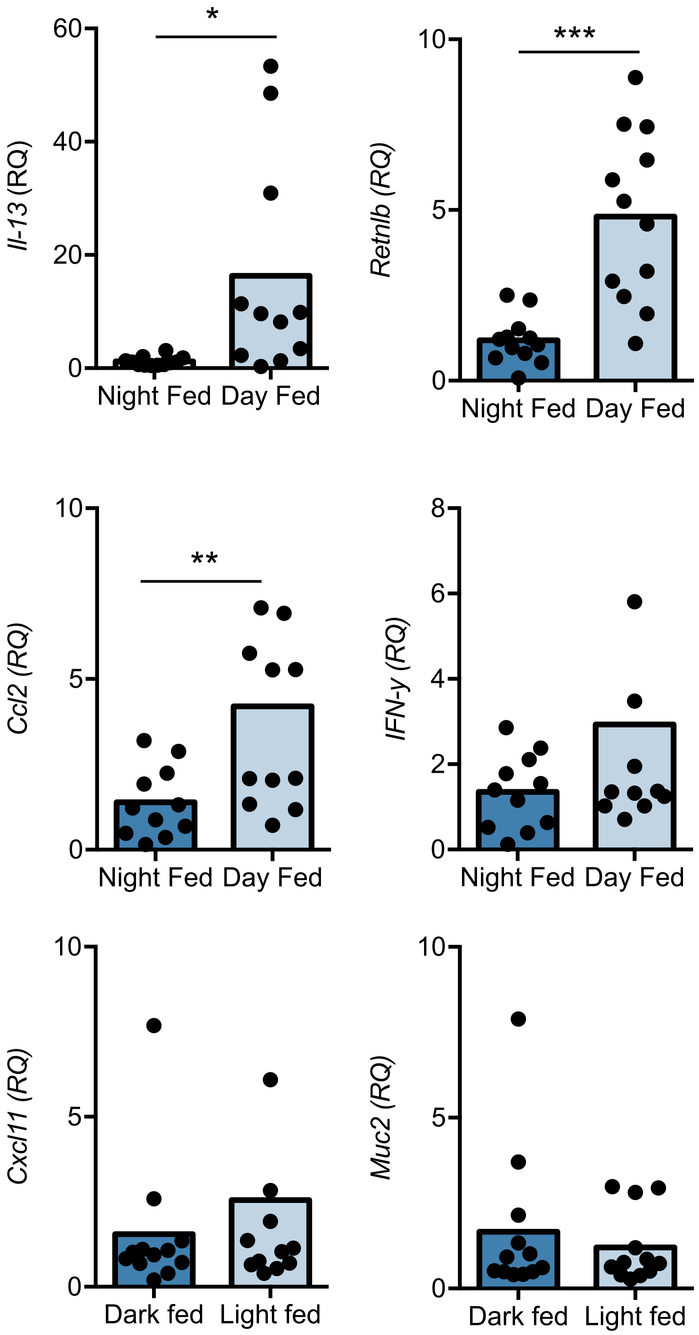

Figure S3: Inflammatory gene expression in gut tissue of mice on restricted feeding regime. Related to Figure 2. Expression of genes in gut tissue harvested from mice on a restricted feeding schedule 21 days post *T. muris* infection, n=11-12, unpaired T-test.

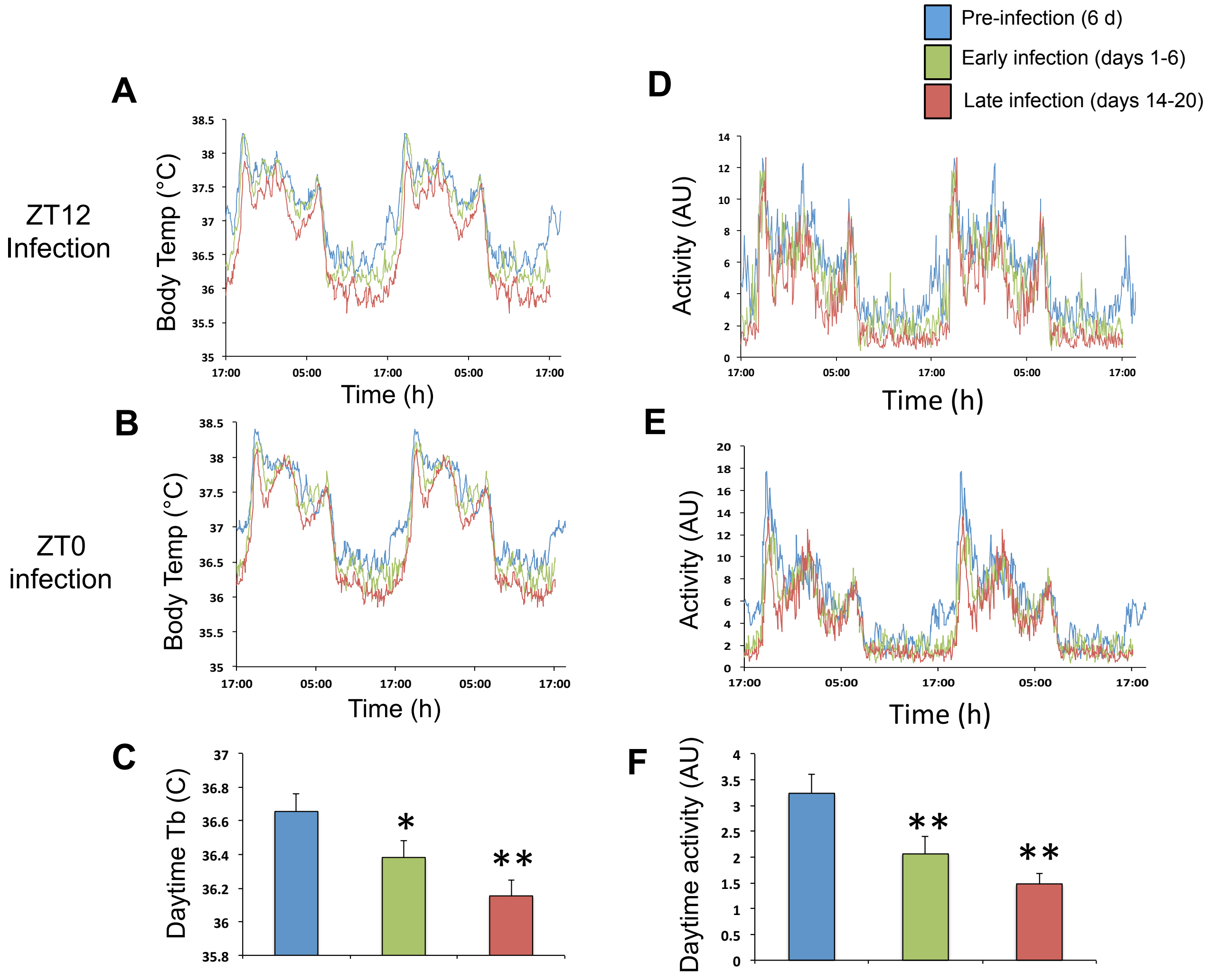

Figure S4: Telemetry recordings from mice infected with *T. muris* eggs. Related to Figure 4.
C57BL/6 mice implanted with telemetry devices 3 days prior to infection with 200 *T. muris* eggs by oral gavage at ZT12 or ZT0, n=6/time point. (A, ZT12 and B, ZT0) Body temperature was recorded in each animal for 6 days prior to infection (Pre - blue), 6 days immediately after infection (Early - green) and for the last 7 days of the study (Late - red). Traces shown are the mean body temperature of all animals in the treatment group averaged for the period of the recordings. Data from mice infected at ZT0 and ZT12 were pooled, as there was no significant effect of infection time on daytime body temperature. (C) There was a significant decrease in basal daytime temperature after infection (One Way ANOVA and post Hoc Tukey) (D and E) Locomotor activity was recorded in each animal; traces shown are the mean activity of all animals in ZT12 infection (D) and ZT0 infection (E). (F) Quantification of daytime activity (pooling ZT0 and ZT12 infected animals) showed decreased activity with *T. muris* infection as measured area under the trace (AU) (One Way ANOVA and post Hoc Tukey).

A

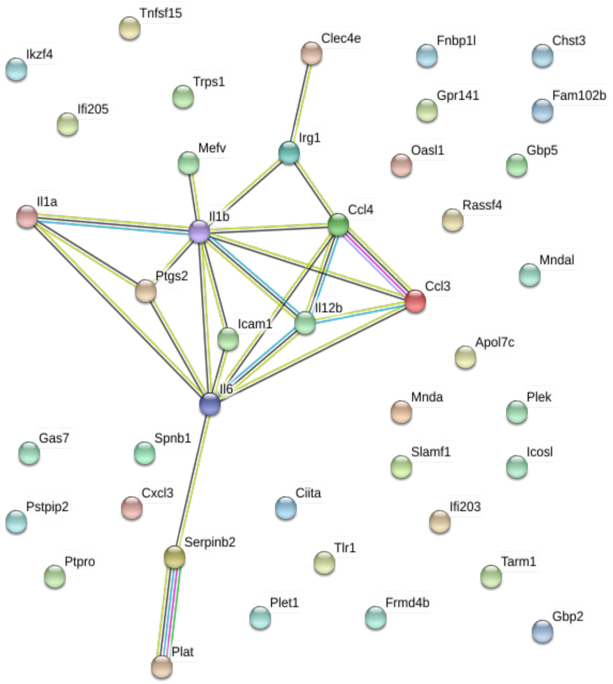

B

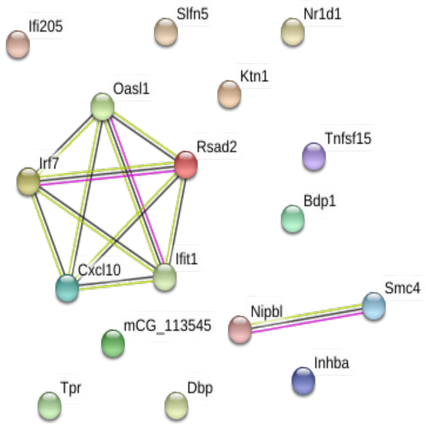

Figure S5: String analysis of RNA Seq data. Related to Figure 6.

(A) Genes that were differentially regulated between *bmal1*$^{fl/fl}$ DCs stimulated by ES antigen at the peak and trough of PER2::luc activity were determined (43 genes) and analysed using the String database. Genes included in the String analysis were extended from Table S2 to include genes with a |Fc|>1.5 and P-adjusted values < 10^{-6}, totalling 43 genes. The figure shows the interactions that were identified. 13/43 genes form a cluster. The probability of finding a set of interacting proteins this large within a set of 43 genes is less the 10^{-14}. (B) Genes that were differentially regulated between *bmal 1*$^{fl/fl}$ and *bmal 1*$^{-/-}$ DC stimulated by ES antigen at trough of PER2::luc activity were determined (17 genes) and analysed using the String database. The figure shows two clusters (one of 5 genes and one of 2 genes). The probability of finding a set of interacting proteins this large within a set of 17 genes is calculated to be less the 10^{-14}.

Table S1: Cellular identity in mesenteric lymph nodes collected 11 days post infection from mice restricted to day or night feeding. Relates to Figure 3.

Values are mean ± SEM, n=5/group. T Tests

Cell type	Night Fed	Day Fed	T-test
Total MLN cells	3.84±0.32	4.34±0.32	NS
Neutrophils (%)	0.36±0.05	0.2906±0.12	NS
B cells (%)	27.24±3.27	35.8±2.29	NS
NK cells (%)	0.354±0.96	0.49±0.07	NS
NKT cells (%)	21.72±4.28	17.88±2.00	NS
T cells (%)	15.368±1.57	22.82±1.69	• P=0.0121
$CD4^+$ T cells (%)	6.64±0.76	10.42±0.81	** P=0.0092
$CD8^+$ T cells (%)	6.70±0.83	10.35±0.87	* P=0.0163
Dendritic Cells (%)	0.514±0.09	0.716±0.03	NS
CD11b hi CD103- DCs (%)	0.109±0.02	0.204±0.01	*** P=0.0041
CD11b+ cd103+ DCs (%)	0.1356±0.02	0.238±0.02	* P=0.0130
CD11b- cd103+ DCs (%)	0.0706±0.01	0.083±0.01	NS

Table S2: Top 20 genes (ranked on Padj) showing significant difference between wildtype DCs stimulated with ES antigen at the peak versus trough of PER2::luc activity, cut of FC>1 or <-1. * Indicates also altered at the trough between CD11c-bmal$^{-/-}$ and Bmal$^{fl/fl}$ after antigen challenge. Relates to Figure 6.

Rank	Gene	Padj	Fold Change	Function
1	*Ptgs2**	5.26E-54	-2.39	Prostaglandin biosynthesis
2	*Apol7c*	1.44E-42	-2.16	Lipid binding
3	*Gas7*	4.9E-32	-2.28	Cell cycle arrest
4	*Ccl3*	3.7E-31	-1.77	Neutrophil chemokine
5	*Il1b*	3.49E-29	-1.61	Cytokine driving IL-12 secretion in dendritic cells
6	*Plet1*	4.74E-25	-2.02	Control of dendritic cell migration via detachment from ECM in intestine [S1]
7	*Mnda*	1.58E-22	-2.15	Transcriptional regulator in myeloid cells
8	*Il12b**	2.3E-22	-2.00	Th1 promoting cytokine
9	*Cxcl3*	3.05E-22	-2.19	Dendritic cell chemokine
10	*Icosl*	3.49E-21	-1.59	Co-stimulatory receptor for T cell activation
11	*Ifi205*	7.42E-21	-3.66	Regulates inflammasome activation
12	*Slc7a2*	1.38E-19	-1.39	APC transporter for arginine and lysine
13	*Tnfsf15**	2.53E-19	-2.90	Driver of T cell production and Th1 cell activation
14	*Irg1*	6.96E-19	-2.21	Negative regulator or TLRs
15	*Ikzf4*	8.88E-19	-1.82	
16	*Icam1*	2.24E-18	-1.58	Adhesion molecule promoting leukocyte migration
17	*Ccl22*	5.33E-18	-1.39	Chemokine for Tregs
18	*Gpr141*	7.39E-18	-1.94	
19	*Clec4e*	1.64E-17	-1.78	
20	*Plat*	3.74E-17	-1.54	Serine protease

Table S3: Top 20 genes (ranked on Padj) showing significant difference between CD11c-bmal$^{-/-}$ versus Bmal$^{fl/fl}$ dendritic cells challenged with ES antigen at the trough of PER2::luc activity, cut of FC>1 or <-1. Relates to Figure 6.

* Indicates also altered in Bmal$^{fl/fl}$ peak

Rank	Gene	Padj	Fold change	Function
1	*Irf7*	5.95E-23	-2.25	Drives Th1 response
2	*Nr1d1*	2.72E-18	-4.40	Circadian
3	*Slfn5*	5.17E-18	-2.02	
4	*Oasl1*	4.69E-14	-2.40	Inhibits translation of IRF7
5	*Dbp*	2.15E-13	-1.83	Circadian
6	*Ptgs2*	7.01E-13	-1.47	COX represses DC immunity ?
7	*Tpr*	7.59E-13	-1.51	
8	*Tnfsf15**	4.32E-12	-2.09	Induces pro-inflammatory cytokines from T cells
9	*Jhdm1d*	7.24E-11	-1.37	Histone demethylase
10	*Cep350*	7.80E-11	-1.38	
11	*Cxcl10*	1.59E-10	-2.00	Optimised Th1 mediated immune response
12	*Ktn1*	1.34E-09	-1.50	Membrane protein
13	*Ifi205*	1.52E-09	-2.23	Interferon activated gene 205
14	*Rsad2*	2.1E-09	-1.975	Promotes IFNγ production in pDCs
15	*Ifit1*	2.92E-09	-3.63	
16	*Gm8995*	3.22E-09	-1.55	
17	*Il12b**	3.22E-09	-1.43	Development of IFNγ secreting Th1 cells
18	*Cry1*	7.60E-09	1.47	Circadian
19	*Ramp1*	8.40E-09	1.48	
20	*Nipbl*	1.31E-08	-1.5	

Supplemental References

[S1] Karrich et al. (2015). Plet1-mediated cell detachment controls steady state migration of dendritic cells in the intestine. International Congress of Mucosal Immunology meeting (Abstract)
